# Targeting CD28, CTLA-4 and PD-L1 Costimulation Differentially Controls Immune Synapses and Function of Human Regulatory and Conventional T-Cells

**DOI:** 10.1371/journal.pone.0083139

**Published:** 2013-12-23

**Authors:** Nahzli Dilek, Nicolas Poirier, Philippe Hulin, Flora Coulon, Caroline Mary, Simon Ville, Henri Vie, Béatrice Clémenceau, Gilles Blancho, Bernard Vanhove

**Affiliations:** 1 Institut National de la Santé Et de la Recherche Médicale, Unité mixte de Recherche 1064, Nantes, France; 2 Institut National de la Santé Et de la Recherche Médicale, Unité mixte de Recherche 892, Nantes, France; 3 Cellular and Tissular Imaging Core Facility (MicroPICell), Nantes, France; 4 Effimune S.A.S, Nantes, France; 5 Centre Hospitalier Universitaire de Nantes, Institut de Transplantation Urologie Néphrologie, Nantes, France; 6 Université de Nantes, Faculté de Médecine, Nantes, France; Institut Pasteur, France

## Abstract

CD28, CTLA-4 and PD-L1, the three identified ligands for CD80/86, are pivotal positive and negative costimulatory molecules that, among other functions, control T cell motility and formation of immune synapse between T cells and antigen-presenting cells (APCs). What remains incompletely understood is how CD28 leads to the activation of effector T cells (Teff) but inhibition of suppression by regulatory T cells (Tregs), while CTLA-4 and PD-L1 inhibit Teff function but are crucial for the suppressive function of Tregs. Using alloreactive human T cells and blocking antibodies, we show here by live cell dynamic microscopy that CD28, CTLA-4, and PD-L1 differentially control velocity, motility and immune synapse formation in activated Teff versus Tregs. Selectively antagonizing CD28 costimulation increased Treg dwell time with APCs and induced calcium mobilization which translated in increased Treg suppressive activity, in contrast with the dampening effect on Teff responses. The increase in Treg suppressive activity after CD28 blockade was also confirmed with polyclonal Tregs. Whereas CTLA-4 played a critical role in Teff by reversing TCR-induced STOP signals, it failed to affect motility in Tregs but was essential for formation of the Treg immune synapse. Furthermore, we identified a novel role for PD-L1-CD80 interactions in suppressing motility specifically in Tregs. Thus, our findings reveal that the three identified ligands of CD80/86, CD28, CTLA-4 and PD-L1, differentially control immune synapse formation and function of the human Teff and Treg cells analyzed here. Individually targeting CD28, CTLA-4 and PD-L1 might therefore represent a valuable therapeutic strategy to treat immune disorders where effector and regulatory T cell functions need to be differentially targeted.

## Introduction

The interaction of CD80/86 and their receptors are important co-stimulatory and co-inhibitory pathways that were shown to regulate Teff responses and peripheral immune tolerance, in particular by controlling Treg development, function and homeostasis. CD28, CTLA-4 and PD-L1 are the three ligands identified on T cells so far that are binding to CD80/86 on human APCs [1], [2], [3]. By controlling T cell motility and activation, these molecules determine whether contacts between conventional effector T cells (Teff) and APCs result in the formation of immunological synapses and in T cell responses [4]. In particular, CD28 and CTLA-4 function like a rheostat to control T cell activation [5]. Costimulation through CD28 in conjunction with triggering of the TCR activates the calcineurin/NF-AT, PKC-θ/NFκB and MAP kinase/AP-1 pathways, leading to production of IL-2 and providing essential survival and proliferation signals to T cells [6], [7]. Resting Teff cells express relatively low levels of CTLA-4 (CD152); however, once activated, T cells increase their membrane expression of CTLA-4, which delivers anti-proliferative signals [8] that block cell cycle transition from G0 to G1 [9] as well as signals leading to inhibition of cytokine production [10] and to Fas-independent cell death [11]. Moreover, CTLA-4 increases T cell motility by inducing T cell polarization and reversing the TCR stop signal [12], [13]. Programmed cell death 1 ligand 1 (PD-L1, also known as CD274 or B7-H1, B7 homolog 1) is inducibly expressed on T cells and can interact with CD80 with an affinity intermediate to that of CD28 and CTLA-4 in humans, resulting in inhibition of T cell proliferation and cytokine production [14]. Furthermore, it has been reported that interactions between Programmed cell death 1 (PD-1) and PD-L1 participate in the maintenance of peripheral tolerance by reducing T cell-dendritic cell (DC) contacts [15].

In addition to their role in Teff activation and survival, costimulatory molecules regulate Treg function and homeostasis. Whereas CD28 signals are critical for Treg cell homeostasis [16], CD28 engagement by CD80/86 inhibits Treg activity [17], [18] presumably via activation of Protein Kinase B/Akt, which inhibits Foxo1 and Foxo3 transcription factors that are required for optimal expression of *Foxp3* and *CTLA4* genes [19], [20]. CTLA-4 itself is required for suppression by Tregs [21] in an intrinsic manner by promoting FoxP3 induction [22], and in an extrinsic manner by inducing IDO in dendritic cells [23] and capturing its CD80/86 ligands from APCs by a process of trans-endocytosis [24]. Murine Tregs are thought to establish LFA-1-dependent cognate contacts and aggregate with CD11c^+^ DCs in vitro or in vivo in lymphoid organs. In contrast with Teff, the contact time of Tregs with APCs is not reduced by CTLA-4 binding to CD80/86 and this may be one explanation for the differential regulation of Teff and Treg responses by CTLA-4 [25]. Whether PD-L1 also regulates Treg-APC contacts has not been clarified yet.

Pharmacological modification of T cell costimulation pathways has become an important therapeutic strategy in autoimmunity, transplantation and cancer. CD80/86 antagonists (Orencia® and Nulojix®) and CTLA-4 antagonists (Yervoy™) are in therapeutic use and antagonists of PD-L1 and CD28 are in development [26], [27]. However, how these agents precisely affect Treg and Teff functions needs deeper investigation. In particular, although these therapeutic strategies emerged from work in rodents more than two decades ago, novel interactions between these costimulatory molecules (for example PD-L1-CD80 interactions) as well as differences between human and rodent costimulation pathways were discovered more recently [4], [28], requiring further characterization of the effects of costimulation in the human setting. In this study, we used T cell clones to study the role and mode of action of antagonist antibodies against CD28, CTLA-4 and PD-L1 on human Teff and Treg responses in vitro. Our data support a ‘mirror-model’ where these CD80/86 ligands exercise an opposite role on the activation and responses of human Teff vs. Tregs.

## Materials and Methods

### Reagents

FR104, a monovalent humanized pegylated Fab’ antibody antagonist of human CD28 [29] was produced by Effimune (Nantes, France). Anti-human CTLA-4 mAb (clone 147.1) was provided by Medarex (Princeton, New-Jersey). Anti-human CD80 (clone M24) and CD86 (clone 1G10) mAbs were provided by Innogenetics (Gent, Belgium). CTLA4-Ig (LEA29Y) was prepared from transfected Cos cells in our laboratory. Anti-CD11a (LFA-1-blocking, clone 38) and anti-PD-L1 (rabbit polyclonal) Abs were purchased from AbD Serotec (Kidlington, UK). Fluorescent mAbs against human CD3 (SP34-2), CD4 (L200), CD25 (M-A251), CD28 (28.6), CD44 (G44-26), CD49d (9F10), CD80 (L307.4), CD86 (2331 (FLW-1)), CD127 (hIL-7R-M21), CTLA-4 (BNI3), ICOS (DX29), ICOS-L (2D3/B7-H2), PD-1 (MIH4), and PD-L1 (MIH1) were from BD Biosciences (San-Diego, California). Fluorescent mAbs against human CD2 (LT2) and Neuropilin-1 (AD5-17F6) were from Miltenyi Biotech (Bergisch Gladbach, Germany). The PE-conjugated anti-human Helios antibody (22F6) was from Biolegend (San Diego, California) and the Alexa 647-conjugated anti-human Foxp3 staining kit (236A/E7) was from eBioscience (San-Diego, California) and used according to the manufacturer’s instructions. Fluorescent mAbs against human CD11a, CD18 (activation epitope) (MEM-148), and CD18 (YFC118.3) were from AbD Serotec. Primary antibodies used for immunohistochemical staining were: rat anti-human CD3 (AbD Serotec) and mouse anti-human PKC-θ (clone 27, BD Bioscience). Fluorescent streptavidin and secondary antibodies were ordered from Life Technologies (Carlsbad, California).

### Generation and Expansion of T Regulatory Clones

CD4^+^CD25^High^CD127^Low^ T regulatory cells from healthy donors were sorted using an Aria flow cytometer (BD Biosciences), plated at 0.3, 1 or 3 cells/well in 96-well plates and stimulated with OKT3 (anti-human CD3 prepared in our lab) in the presence of irradiated (35 Gy) feeder cells (allogeneic peripheral blood mononuclear cells (PBMC) at 6.10^5^ cell/ml and a pool of 3 allogeneic Epstein-Barr Virus (EBV)-transformed B-cell lines at 6.10^4^ cell/ml) for 7 days at 37°C, 5% CO2, in complete medium (RPMI 1640, 8% heat-inactivated pooled human serum, 2 mM L-glutamine, 100 U/ml penicillin, 0.1 mg/ml streptomycin, 1% non-essential amino acids, 1 mM sodium pyruvate and 5 mM Hepes, all from Life Technologies) supplemented with 300 UI/ml IL-2. Starting on day 8, Treg clones were expanded by stimulation with plastic-coated OKT3 and soluble anti-CD28 (CD28.2, from our lab) in the presence of 1,000 UI/ml IL-2 for 14 days. After 21 days, the clones were restimulated once with feeder cells in the presence of IL-2 and OKT3 as described above. The clones were tested for phenotype and function after 6 weeks of clonal expansion.

### Treg Clones Suppression in Mixed Lymphocyte Reactions (MLR)

Peripheral blood mononuclear cells (PBMCs) were isolated from whole blood by density gradient using Ficoll-Paque (PAA, Piscataway, New Jersey). Freshly isolated PBMCs were co-cultured with allogeneic irradiated PBMCs (10^5^ cells/well of each cell type) and autologous CD4^+^CD25^hi^CD127^low^ polyclonal Tregs or Treg clones at a 1∶1 or 1∶1/10 ratio for 5 days at 37°C, 5% CO2 in complete medium. Cells were pulsed with 1 µCi of ^3^H-thymidine during the final 8 hours of culture and then harvested and counted in a scintillation counter.

### Live Cell Dynamic Microscopy

A human allo-specific CD4^+^CD28^+^ T cell clone [30] (Teff; 2.10^5^ cells) or a human T regulatory cell clone (Treg#1; 2.10^5^ cells) were used in their resting state (two weeks after their last stimulation), stained with the FURA-2 AM probe (0.5 µM for 30 min; Interchim, Montluçon, France), washed and added to 4.10^5^ human EBV-transformed B lymphoblastoïd cells, as described [31], on a coverslip coated with Poly-L-Lysine (0.001%; Sigma, Saint-Louis, Minnesota). Bright-field and fluorescent images were acquired at 15 second intervals on a Leica microscope (Leica Microsystems, Wetzler, Germany) using the Metafluor image analysis software (Molecular Devices, Sunnyvale, CA). Individual T cell-APC interactions and individual T cell calcium peaks were recorded manually over a 20 min. incubation period with Metafluor (version 7.1.7) and Metamorph (version 7.5.6; Roper Scientific, Göttingen, Germany) software. A calcium peak was recorded when fluorescence levels reached 2-fold the baseline level. T cells were tracked using the ImageJ free software (version 1.41). Antibodies were all used at 10 µg/ml. Data are presented as mean ± SD for each condition.

### Suppression Assays and Cytokine Release Assays

All experiments were performed with PBMCs obtained from healthy donors and the Treg#1 clone. CD4^+^ T cells were enriched from PBMCs by negative selection using CD4^+^ T cells isolation Kit II (Miltenyi) and an autoMACS Pro separator (Miltenyi). Enriched CD4^+^ cells were then stained with anti-human CD4, CD25 and CD127 mAbs at 4°C for 30 min. CD4^+^CD25^hi^CD127^low^ regulatory T cells (formerly referred to as natural Treg and now recently proposed to be named thymus-derived Treg or tTreg [32]) and CD4^+^CD25^−^ naïve T cells were sorted (purity routinely above 95%) with a high-speed cell sorter (FACSAria; BD Biosciences) and FACSDiva software (BD Biosciences).

Allogeneic mature DC (mDCs) were generated from monocytes as described [33]. Briefly, monocytes were enriched by elutriation (>85% CD14^+^) and cultured for 5 days in medium supplemented with IL-4 (40 ng/ml; R&D Systems, Minneapolis, Minnesota) and GM-CSF (1,000 IU/ml; Gentaur, Kampenhout, Belgium). Cells were harvested on day 5 and cultured for 24 h with LPS to induce maturation (1 µg/ml; *E. coli* 0111:B4, Sigma). Ten thousand allogeneic mature DCs (mDCs) were then co-cultured with 10^5^, 10^4^, 10^3^, or 10^2^ Tregs (nTregs or Treg#1 clone) in complete medium with or without anti-CD28 blocking antibodies (10 µg/ml) for 18 h. mDC/Treg cultures were washed and added to 10^5^ CD4^+^CD25^−^ cells (same donor as nTregs) stimulated with 10^4^ allogeneic mDCs (same donor as mDCs used for Treg activation). Cells were then cultured for 5 additional days and proliferative responses were assessed by ^3^H-thymidine incorporation. After 24 h or 72 h, 50 µL supernatant was collected from each triplicate, pooled and analyzed for IL-2, IL-6, TNF-α, and IFN-γ by BD™ Cytometric Bead Array (CBA) according to the manufacturer’s instructions (Human Th1/Th2 Cytokine Kit II; BD Biosciences).

### Activated LFA-1 and Adhesion Molecules Analyses and Flow Cytometry

Teff and Treg#1 clone (2.10^5^ cells/well for each) were incubated with indicated antibodies (10 µg/ml) during 30 min in HBSS 1X, 5 mM Hepes at 37°C. After washing, they were added to human EBV-transformed B lymphoblastoïd cell lines (pool of cells from 3 donors, 4.10^5^ cells/well) in the presence of fluorescent anti-CD11a, anti-CD18act (MEM-148) or anti-CD18tot (YFC118.3) mAbs and incubated during 30 min in HBSS 1X, 5 mM Hepes at 37°C. After washing, cells were stained with fluorescent anti-CD3 and anti-CD4 antibodies. Alternatively, cells were stained with fluorescent anti-CD2, anti-CD44, anti-CD49d and anti-Nrp1 antibodies. Samples were acquired on a BD FACSCANTO™ flow cytometer (BD Biosciences) and analyzed with the FlowJo software.

### Immunohistochemistry

Teff and Treg#1 clone (4.10^5^ cells/well for each) were incubated with anti-CD28 blocking Fabs (FR104) at 10 µg/ml for 30 min in HBSS 1X, 5 mM Hepes at 37°C and were added to 8.10^5^ human EBV-transformed B lymphoblastoïd cell lines (pool of cells from 3 donors) on a coverslip coated with Poly-L-Lysine (0.01%; Sigma). After incubation for 15 min at 37°C, cells were fixed with paraformaldehyde (PFA) 4% during 20 min at room temperature. Cells were saturated and permeabilized with PBS containing 4% BSA, 2% normal goat serum and 0.1% triton-X100 at RT. Cells were incubated overnight with primary Abs at 4°C, followed by fluorescent secondary Abs and nuclear staining (DAPI; Invitrogen). T-APC interactions were evidenced by a double staining with anti-human CD3 antibodies (followed by goat anti-rat IgG-Alexa 568; Life Technologies) and anti-human PKC-θ (followed by goat anti-mouse IgG-Alexa 488; Life Technologies). Cells were analyzed by confocal fluorescence microscopy (Nikkon, Tokyo, Japan) and the NIS-Element imaging software (Nikon).

### Statistical Analyses

Suppression assay data were analyzed with unpaired t tests (Mann-Whitney). Time-lapse data were analyzed with Kruskal*–*Wallis one-way analysis. *P* values less than 0.05 were considered statistically significant. All statistical analyses were performed on a personal computer with GraphPad InStat (version 5.1, GraphPad Software, San Diego, CA, USA).

## Results

### Phenotype and Suppressive Function Analysis of Human T Regulatory Cell Clones

**Figure 1:**
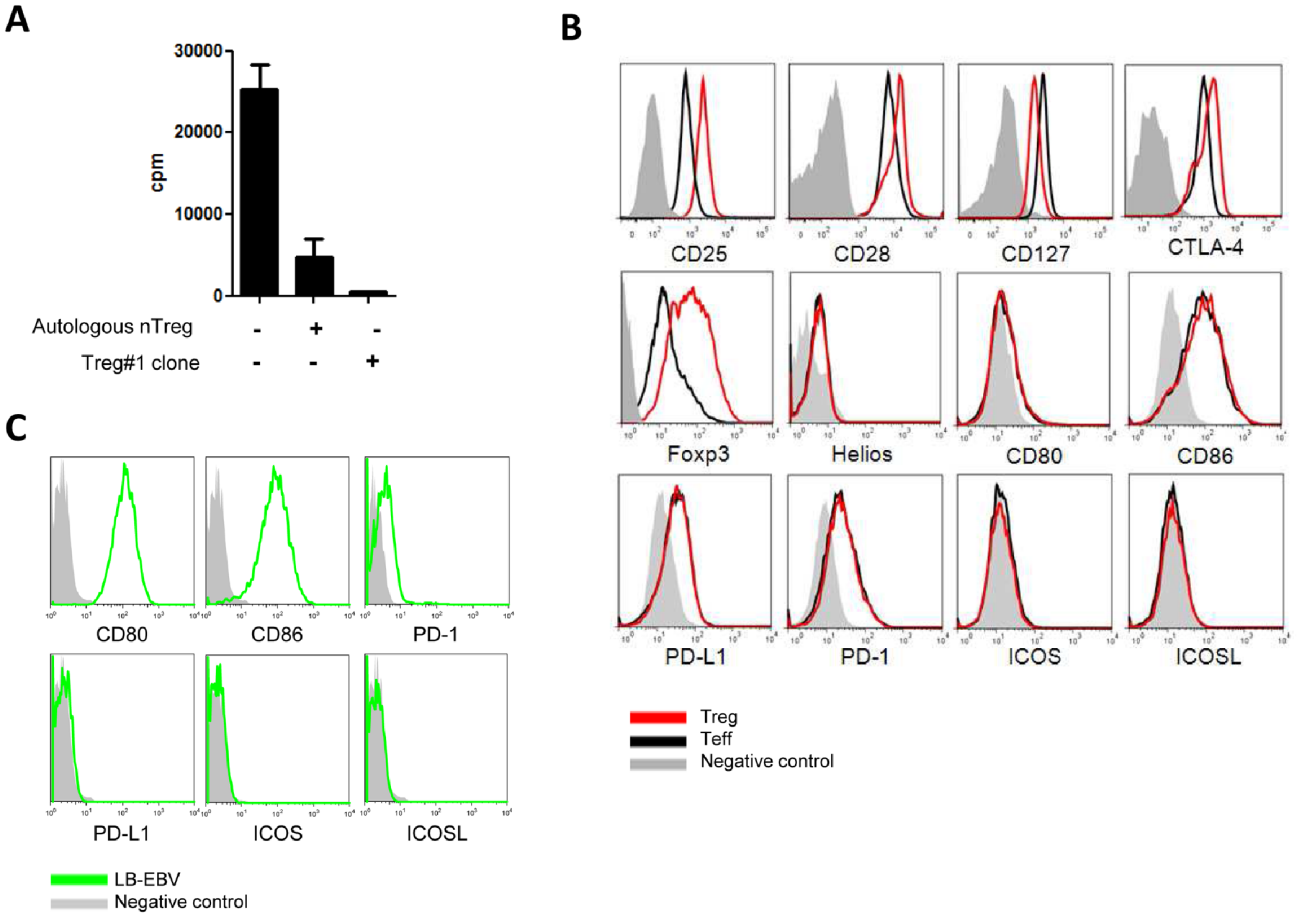
Phenotypic and functional analyses of human regulatory T cells (Treg#1) and allogeneic human B-EBV cells phenotype. (**A**) Proliferation of CD4^+^CD25^−^ T cells stimulated with irradiated allogeneic PBMC at day 5 in presence or not of autologous natural CD4^+^CD25^High^CD127^Low^ regulatory T cells (nTreg) or clone Treg#1 at a 1∶1 ratio. **P<0.01. (**B**) The phenotype of Treg#1 clone is compared to effector T cell (Teff) by Flow Cytometry. Control: filled gray, Teff: black line and Treg#1: red line. (**C**) Costimulatory molecule expression analysis on CD20^+^ B-EBV lymphocytes (pool of 3 cell lines). Control: filled gray and B-EBV cells: green line.

Analyzing contacts between polyclonal T cells and allogeneic APCs using live cell dynamic microscopy is technically challenging since allogeneic cognate contacts occur at a low frequency. Thus, to compare the costimulation requirements of human Tregs and Teff, we used previously described Teff clones [34] and produced new human Treg clones. Both Teff and Treg clones were generated by stimulation of cells from healthy donors by allogeneic B-EBV cells. We first selected 16 Treg clones out of 50 that expressed Foxp3, tested their suppressive function and found that 3 clones presented a suppressive activity in MLR. We selected the clone “Plate 2_A1” (hereafter referred to as Treg#1) for use in the remainder of the study. Clone Treg#1 presented a 98% suppressive activity at a 1∶1 ratio in MLR, which was higher than the 81% suppressive activity of autologous polyclonal nTregs assessed in parallel (Fig. 1A). After expansion, allospecific T cell clones presented two distinct phenotypes: CD4^+^CD25^+^CD28^+^CD127^hi^CTLA-4^+^Foxp3^−^ for Teff and CD4^+^CD25^hi^CD28^hi^CD127^low^CTLA-4^hi^Foxp3^+^ for Tregs (Fig. 1B). Both clones were negative for Helios, CD80, ICOS and ICOS-L and similarly expressed CD86, PD-1 and PD-L1 costimulatory molecules (Fig. 1B). Our Teff cells thus presented a phenotype of primed/activated T cells and our Treg cells presented a phenotype similar to Helios-negative peripheral tTreg. The EBV-transformed B lymphoblastoïd cell lines used here expressed CD80 and CD86 but did not express CTLA-4, PD-1, PD-L1, ICOS and ICOS-L costimulatory molecules (Fig. 1C).

### Control of Teff Synapses, Motility, Velocity and Activation by CD80/86 Ligands

**Figure 2:**
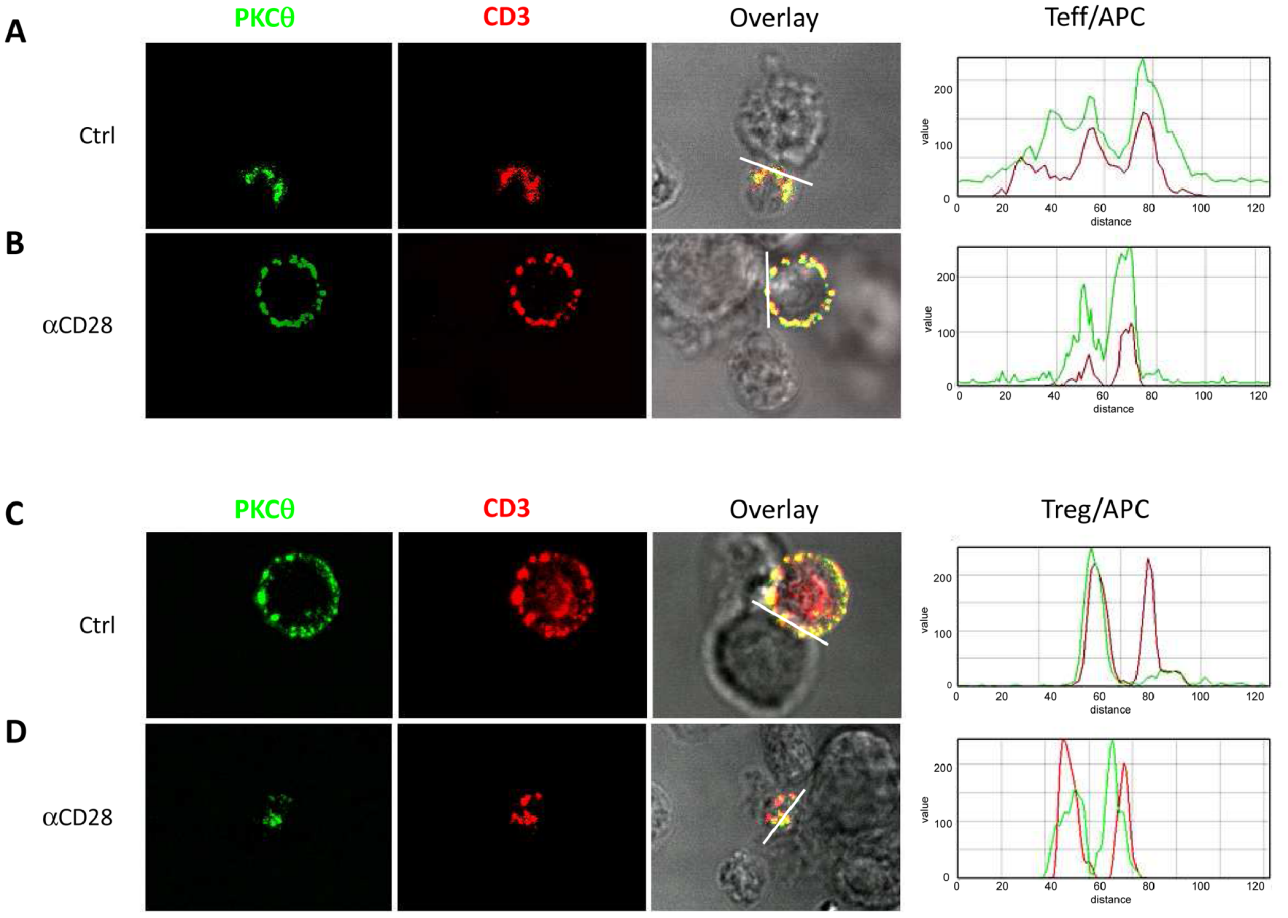
Selective-CD28 blockade breaks Teff/APC immunological synapses (IS) and enhances Treg/APC immunological synapses. Human Teff and Treg were introduced into Labtech coated with poly-L-lysine and containing a pool of 3 B-EBV cell lines (APC). After 15 min incubation at 37°C, cells were fixed, stained and imaged by confocal microscopy. (**A**) Localization of CD3 (red) and PKC-θ (green) in the IS formed by Teff and APCs. (**B**) Expression of CD3 and PKC-θ on whole cell after CD28-blockade. (**C**) Distribution of CD3 (red) and PKC-θ (green) on Treg cells in control condition. (**D**) Polarization of CD3 and PKC-θ in the IS formed by Treg and APCs after CD28-blockade. Ten to 15 microscope fields were examined and the experiment has been repeated 3 times independently Therefore data are representative of more than 90 events. Histograms represent CD3 (red) and PKC-θ (green) intensity at the interaction between Teff (A, B) and Treg (C, D) with APCs and indicate the distribution of these 2 molecules at the interaction level with or without CD28 blocking antibody.

**Figure 3:**
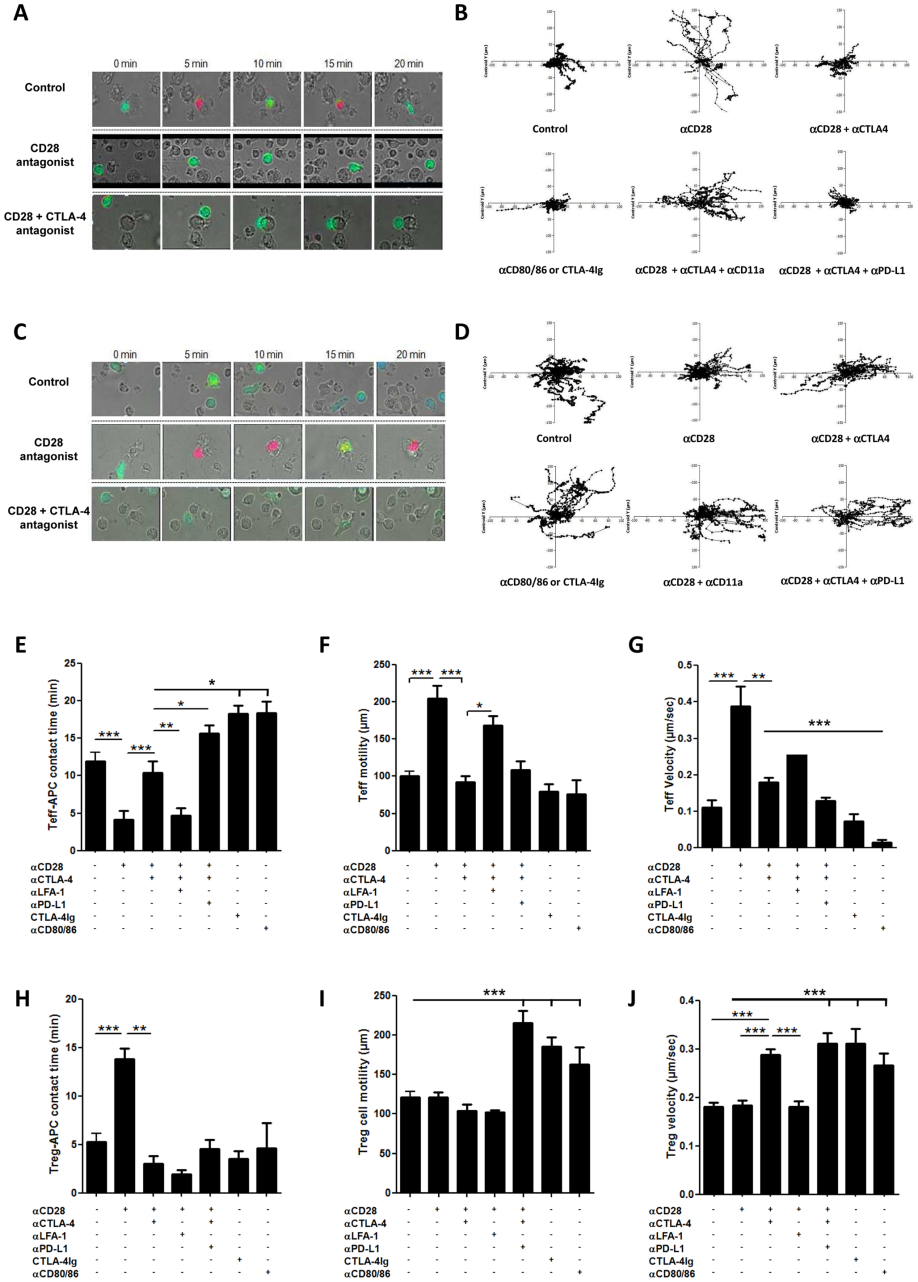
Live-cell dynamic analysis of human Teff and Treg cells in presence of human B-EBV APCs. (**A, C**) Screenshots from movies representing Teff or Treg cells stained in green (non-activated cells) or red (activated cells) by Fura2-AM calcium probe in the presence of unstained B-EBV lymphocytes (APCs) in poly-L-lysine coated Labtech. Teff and Treg were pre-incubated with anti-CD28 (FR104) or anti-CD28+ anti-CTLA-4 or nothing (control). Images were analyzed over a period of 20 min. (**B, D**) Teff and Treg cell motility over 20 minutes period in indicated conditions. (**E**) Mean of contact times between Teff cells and APCs in the presence or not of various indicated antibodies. (**F**) Mean of traveled distances by Teff over 20 minutes in the same conditions as above. (**G**) Mean of Teff cell velocity over 20 minutes in the same conditions as above. (**H**) Mean of contact times between Treg cells and APCs in the presence or not of various indicated antibodies. (**I**) Mean of traveled distances by Treg cells over 20 minutes in the same conditions as above. (**J**) Measurement of Teff cell velocity over 20 minute period in the same conditions as above. All indicated antibodies were used at 10 µg/ml. (n≥30 cells for each condition) ***P<0.001; **P<0.01 and *P<0.05.

By using Teff clones and their cognate allogeneic B cells, it was possible to capture several antigen-specific contact events per microscope field. Their antigen-specificity was confirmed by the observation that no contacts formed in the presence of another B-cell line (Raji cells; data not shown). Teff cells in their resting state were incubated with their cognate allogeneic B cells in the presence or absence of blocking reagents for different costimulation pathways, and T cell-APC contacts as well as T cell motility and activation were measured. Cells were added onto poly-L-lysin coated slides that allow mild but free cell movements. To assess the CD28 dependency of T cell motility and synapse formation, we used FR104, a novel anti-CD28 antagonist monovalent antibody that induces immunosuppression by blocking CD28-mediated costimulation with a demonstrated absence of signaling activity [29], [35]. We first controlled that contacts between Teff and APCs actually resulted in formation of true immunological synapses (IS) in our system, defined by recruitment and colocalization of CD3 and PKC-θ at the interaction site. As expected, interaction of allospecific Teff with allogeneic B cells used as APCs resulted in the formation of IS, and this was prevented by CD28 blockade (Fig. 2A, B). In addition, the dwell time of Teff on APCs was strongly reduced by CD28 blockade (4.16±1.19 min. with FR104 during a 20-min observation period *vs* 11.93±1.17 min. without FR104, p<0.001; Fig. 3A, 3E and Movies S1, S2). The effect of CD28 blockade on conjugate formation was abolished by the simultaneous blockade of CTLA-4 (dwell time: 10.45±1.53 min. with FR104+ anti-CTLA-4 vs. 4.16±1.19 min. with FR104 alone; Fig. 3A, 3E and Movie S3), suggesting that engagement of CTLA-4 dominantly induces reversal of the TCR-STOP signal in the absence of CD28 costimulation and that concomitant CD28 and CTLA-4 signals balance each other out to regulate the duration of Teff contacts to APCs. In contrast, CTLA-4Ig or anti-CD80/86 antibodies significantly prolonged Teff-APC contact time as compared to control (dwell time: 18.33±1.07 min. and 18.42±1.54 min., respectively, vs. 11.93±1.17 min. for control; Fig. 3E). The fact that CTLA-4Ig or anti-CD80/86 antibodies induced higher contact times than combined antagonists for CD28 and CTLA-4 suggested that another ligand of CD80/86 might also repress dwell time. A likely candidate is PD-L1 since it is the third described ligand of CD80, besides CD28 and CTLA-4 [1]. In agreement with this hypothesis, addition of anti-PD-L1 antagonist mAbs to CD28 and CTLA-4 antagonists recapitulated the effect of CTLA4-Ig or anti-CD80/86 mAbs on contact times of Teff with APCs (15.67±1.13 min, not significantly different from the contact time obtained with CTLA4-Ig or anti-CD80/86 mAbs; Fig. 3E). Of note, the fact that the B cell line used as APC in this assay did not express PD-L1 (Fig. 1C) indicates that the effect of anti-PD-L1 antagonists is due to the blockade of PD-L1 on T cells. Interestingly, Teff cells morphology changes induced by APCs were much less important in the presence of anti-CD28 plus anti-CTLA-4 mAbs, or in the presence of CTLA4Ig (Movie S3), than in control conditions or than in the presence of anti-CD28 mAbs alone, suggesting a control of cell morphology by CTLA-4.

The motility and velocity of Teff inversely correlated with dwell time, as they increased in the presence of FR104 (from 100.5±6.03 µm to 204.8±17.54 µm, p<0.001, for motility and 0.11±0.02 to 0.39±0.05 µm/sec, p<0.01, for velocity). When anti-CTLA-4 mAbs were added to FR104, motility and velocity were restored to levels similar to controls (92.42±7.68 µm and 0.18±0.01 µm/sec, respectively; Fig. 3B, F, G). CTLA-4Ig and anti-CD80/86 antibodies induced no modification of motility (79.12±10.32 µm and 75.68±19.19 µm, respectively) and a non significant tendency toward reduced velocity (0.07±0.02 µm/sec and 0.014±0.007 µm/sec, respectively; Fig. 3B, F).

Calcium flux analyses, reporting on the number of calcium peaks per minute, showed decreased calcium flux in Teff in the presence of CD28 antagonists (0.36±0.03 peaks/min in control conditions vs. 0.15±0.02 peaks/min with FR104, p<0.001; Fig. 3A, B). Although a minority of T cells still established prolonged contacts with APCs in the presence of CD28 antagonists, these contacts resulted in reduced calcium influx on a per cell basis as compared to controls. In contrast with dwell time, motility and velocity parameters, the reduction of calcium flux by CD28 antagonists was not reversed by CTLA-4 blockade (0.15±0.02 peaks/min in the presence of FR104, with or without CTLA-4 blockade). In addition, incubation with CTLA4-Ig or CD80/86 antibodies was as efficient as CD28 antagonists to inhibit calcium flux (0.08±0.03 and 0.11±0.02 peaks/min, respectively; Fig. 3B). These data highlight that whereas Teff motility and contact formation are influenced by CTLA-4 even in the absence of CD28 signals, Teff activation (measured by calcium flux) is abrogated in the absence of CD28 signaling regardless of CTLA-4 engagement.

### Enhancement of Synapses, Motility and Activation in Tregs by CD80/86 Ligands

We have previously shown that CD28 antagonists promoted suppressive activity of polyclonal Treg cells *in vitro* and *in vivo* [34]. In this study, we analyzed whether this effect could be related to parameters of allogeneic Treg contact with APCs at the single-cell level. Like for Teff, the clonal Treg cells used here were not able to form cognate contacts in the presence of other APCs than the allogeneic B-EBV lymphoblastoid cells used for their amplification (data not shown). In control conditions, these Treg cells established only short contacts with their allogeneic APCs in comparison with Teff (5.25±0.87 min for Tregs vs. 11.93±1.17 min. for Teff). In contrast, Treg-APC contacts were prolonged in the presence of CD28 antagonists (13.81±1.10 min with FR104 vs. 5.25±0.87 min without, p<0.001; Fig. 3C, 3H and Movies S4, S5) and their duration became similar to Teff-APC contact times assessed in control conditions. These extended contact times after CD28 blockade were dependent on CTLA-4 availability since addition of anti-CTLA-4 mAbs totally reversed the effect of the CD28 antagonists on Treg-APC contact times (3.02±0.82 min with FR104 and anti-CTLA-4 vs. 13.81±1.10 min with FR104 alone; Fig. 3H, Movie S6). Thus, CTLA-4 controls contact formation between these human Tregs and APCs in the absence of CD28 signaling. Direct blockade of CD80/86 with CTLA4-Ig or anti-CD80/86 mAbs, which prevents engagement of both CD28 and CTLA-4, did not significantly change the time during which Tregs dwelled on APCs (3.50±0.83 min and 4.64±2.59 min, respectively, vs. 5.25±0.87 min in controls; Fig. 3H).

CD28 antagonists, with or without concomitant CTLA-4 blockade, did not alter the motility of Tregs compared to controls (Fig. 3D, I). The fact that CD28 antagonists increased Treg-APC contact times without decreasing Treg motility could be explained by the observation that Treg-APC moved together in pairs after initial contact was established (data not shown), a phenomenon that we did not record for Teff. Surprisingly, CTLA4-Ig and anti-CD80/86 mAbs induced a significant increase in Treg motility (186.0±11.94 µm and 163.0±21.87 µm, respectively, vs. 121.7±6.98 in controls, p<0.01) (Fig. 3D, I). As above, the differential effect of CD28/CTLA-4 vs. CD80/86 blockade on Treg motility suggested a role for the PD-L1/CD80 interaction. Indeed, a striking difference in Treg motility was noticed after addition of a PD-L1 antagonist to CD28 plus CTLA-4 blocking reagents (104.3±7.61 after CD28+ CTLA-4 blockade vs. 215.4±15.86 µm after CD28+ CTLA-4+ PD-L1 blockade). This data demonstrate for the first time a direct effect of PD-L1 on motility of a Treg clone, and thus uncovers a novel function for PD-L1/CD80 interactions (PD-L1 in our system cannot interact with PD-1 since B cells used here as APCs do not express PD-1). Contrary to Teff cells, CD28 blockade alone had no effect on Treg velocity (Fig. 3J). However, the simultaneous blockade of CD28 and CTLA-4, or the use of CTLA4-Ig or anti-CD80/86 mAbs significantly increased Treg velocity (0.29±0.01 µm/sec, 0.31±0.03 µm/sec or 0.27±0.02 µm/sec, respectively, vs. 0.18±0.05 in controls) (Fig. 3J).

**Figure 4:**
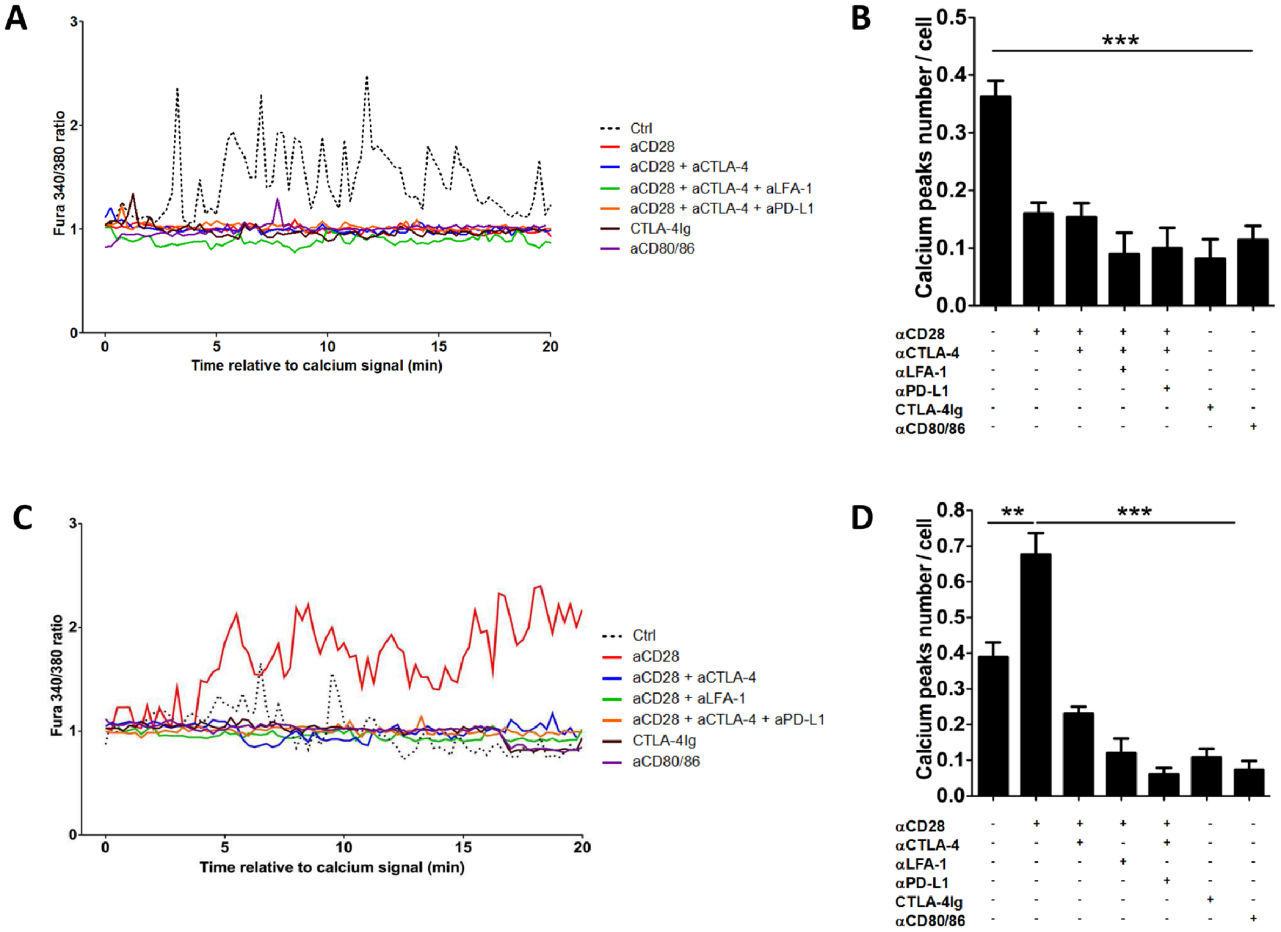
Calcium flux profiles and quantification of calcium responses in Teff and Treg. The calcium flux of Teff (**A**) and Treg (**C**) cells, which established contacts with APCs, were analyzed by measuring the fluorescence of calcium probe (Fura2-AM) over a period of 20 minutes. All antibodies were used at 10 µg/ml. The profile of one representative cell for each condition is shown. Quantification of Teff cell (**B**) and Treg (**D**) activation was set by the number of calcium flux peaks/min. The number of calcium peaks analyzed in each condition was >30. *P<0.05 and ***p<0.001.

Analysis of calcium flux in control conditions revealed a low activation level in Tregs compared with Teff. Actually the number of calcium peaks per minute in Treg was not different but of much lower intensity than in Teff, (Fig. 4). Interruption of CD28-mediated signals with FR104 resulted in a significant increase in calcium mobilization in Tregs (the number of calcium peaks per minute raised from 0.39±0.04 peaks/min in control condition to 0.68±0.06 peaks/min with FR104, p<0.05; Fig. 4D), that was mostly visible after a 5 min incubation period (Fig. 4C). In accordance with data showing that CTLA-4 is essential for Treg suppressive function [36], we observed that concomitant blockade of CTLA-4 signals inhibited Treg calcium responses (0.23±0.02 peaks/min with FR104+ anti-CTLA-4 vs. 0.68±0.06 peaks/min with FR104 alone). Blockade of CD80/86 resulted in even stronger inhibition of calcium flux than blockade of both CD28 and CTLA-4 (0.07±0.02 peaks/min or 0.11±0.02 peaks/min with anti-CD80/86 antibodies or CTLA-4Ig, respectively; Fig. 4D). Taken together, these data indicate that in contrast with Teff, CD28 blockade in these experiments increased Treg-APC contacts and Treg activation, and that these mechanisms were dependent on CTLA-4.

Finally, as with Teff cells, confocal microscopy was performed to determine the formation of IS in Tregs. In contrast with a previous report showing an opposite localization of PKC-θ from the IS and no colocalization between CD3 and PKC-θ in human Tregs [37], we observed that stimulation of human Treg cells induced some colocalization of CD3 and PKC-θ molecules over the entire cell membrane without cluster formation at the interaction side (Fig. 2C). When CD28-mediated signaling was inhibited, clusters of CD3 molecules became apparent and were colocalized with PKC-θ at the Treg-APC interface (Fig. 2D), which is compatible with the formation of a stable IS and the calcium mobilization observed in these conditions.

### LFA-1-dependent Teff-APC and Treg-APC Contacts

**Figure 5:**
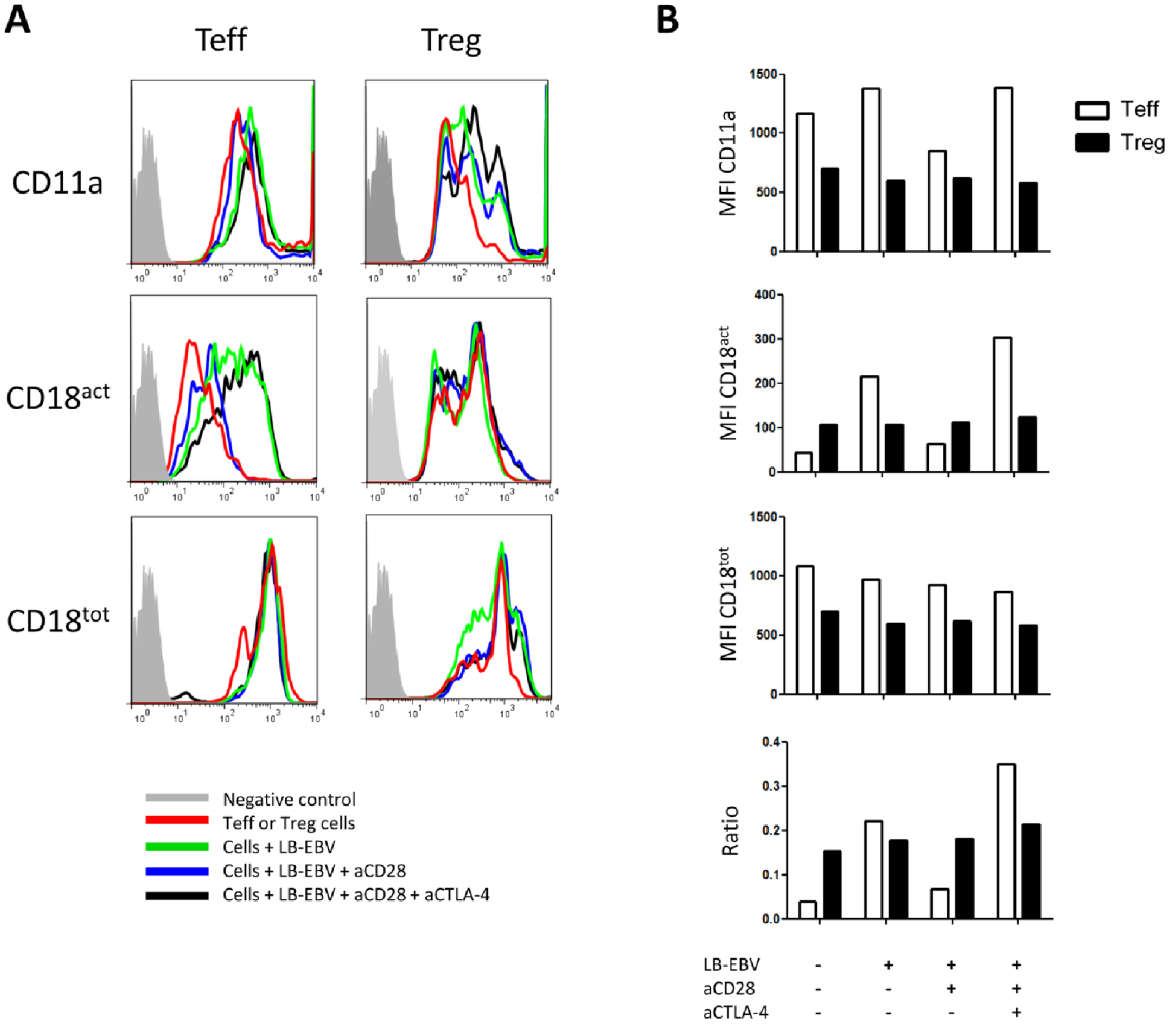
LFA-1 activation analyses by Flow Cytometry. (**A**) Profiles of CD11a, CD18 activation epitope (CD18^act^, representing “high affinity” conformation) and CD18 (CD18^tot^) expression by Teff and Treg#1 cells pre-incubated or not with indicated antibodies. Anti-CD28 and anti-CTLA-4 were used at 10 µg/ml. (**B**) Histograms of Mean Fluorescent Intensity (MFI) of CD11a, CD18^act^ and CD18^tot^ expressed on Teff and Treg#1 cells. Ratio CD18^act^ MFI: CD18^tot^ was established to analyze LFA-1 high affinity conformation in indicated conditions. Data are representative of more than three different experiments. Filled gray, negative control; Red line, Teff and Treg cells alone; Green line, cells with APC; Blue line, cells with APC and anti-CD28; and Black line: cells with APC, anti-CD28 and anti-CTLA-4.

The integrin LFA-1 (CD11a/CD18) is a well-established intercellular adhesion molecule that plays a key role in several stages of T cell activation and effector function [38]. The LFA-1 molecule exists in three conformations (low, intermediate and high affinity) and is important for the formation of IS [39], [40], especially in its high affinity conformation. Therefore, we analyzed LFA-1 expression and conformation and its relationship with T cell function in our Teff and Treg clones. CD11a and CD18 were constitutively expressed by Tregs and Teff (Fig. 5). However, while the constitutive expression of CD11a and CD18 was lower in Tregs compared to Teff, the high affinity conformation of LFA-1 (detected by staining for CD18 activation epitope) was constitutively expressed at higher levels in Tregs compared to Teff. As expected, the ratio of LFA-1 of the high affinity conformation over total LFA-1 was increased when Teff cells were added to APCs (0.039 in Teff alone vs. 0.221 in the presence of APCs; Fig. 5A, B). Interestingly, this ratio was decreased by 3.25-fold after addition of a CD28 antagonist, a phenomenon completely reversed by the simultaneous blockade of CTLA-4 (ratio = 0.35; Fig. 5A, B). This suggested that signaling through CD28 favored whereas CTLA-4 inhibited expression of the high affinity conformation of LFA-1 in these Teff cells. In contrast, the constitutively high expression of the activated form of LFA-1 in Tregs was stable and not influenced by addition of APCs or inhibition of CD28 and CTLA-4 signals (Fig. 5A, B).

Next, we tested the effect of LFA-1 blockade on stable T cell-APC interactions by live-cell dynamic microscopy. The addition of anti-CD11a antagonist mAbs to Teff in conditions of high contact times and low motility (i.e. conditions where CD28 and CTLA-4 were blocked) shortened Teff-APC contacts (4.72±0.95 min vs. 10.45±1.53 min; Fig. 3E) and increased Teff motility and velocity (168.3±12.46 µm and 0.28±0.02 µm/sec, respectively; Fig. 3F, G). In Tregs, LFA-1 blockade abrogated the long lasting contacts induced by CD28 antagonists (1.96±0.38 min vs. 13.81±1.10 min., p<0.001; Fig. 3H) without affecting motility and velocity (102.2±3.04 µm and 0.18±0.01 µm/sec, respectively; Fig. 3I, J). Accordingly, LFA-1 blockade reversed the increased calcium peaks induced by CD28 antagonists in Tregs (Fig. 4D). Together, these results confirmed a role of LFA-1 in Teff/APCs and Treg/APCs dwell time and revealed a role for LFA-1 in the activation of Treg. Analyses of other adhesion molecules, such as CD2, Neuropilin 1 (Nrp1), CD44 and CD49d did not reveal significant differences in expression level on human Teff and Treg clones in any conditions tested here (data not shown).

### Enhancement of Treg Suppressive Function by CD28 Blockade

**Figure 6:**
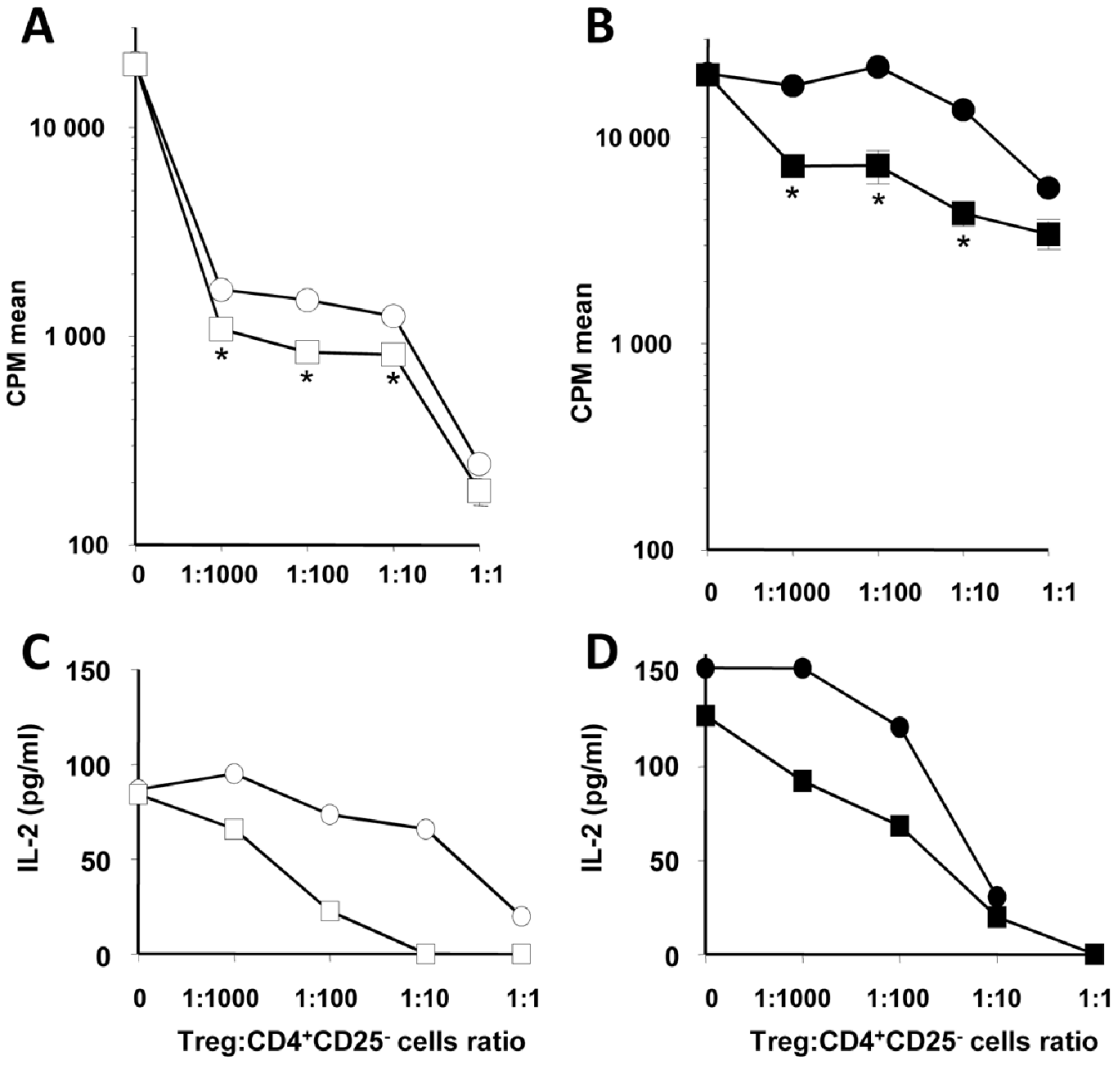
Enhanced suppressive activity of Treg with selective CD28-blockade. Treg cells were first primed against alloantigens, washed out and added to CD4^+^CD25^−^ effector T cells stimulated with allogeneic irradiated mDC at the indicated ratio. Priming was performed with or without CD28 antagonists. Round symbols: no antagonist. Squares: addition of a CD28 antagonist. CD4^+^CD25^−^ effector T cells proliferation (A, B) and IL-2 synthesis (C, D) after addition of clonal Treg (empty symbols; A, C) or natural Treg (filled symbols; B, D). Addition of Treg to APC in the absence of CD4^+^CD25^−^ effector T cells resulted in less than 1000 CPM and undetectable IL-2 synthesis (data not shown). Results are mean cpm ± SD or concentration in supernatants measured by CBA of one representative assay out of 3. *p<0.05.

To understand whether modifications of Treg-APC contacts and Treg motility or activation induced by costimulatory signals translated into a parallel modification of function, we measured Treg activity in suppression assays in the presence of antagonists for individual costimulation pathways. Tregs were first primed in the presence of allogeneic mDCs with or without CD28 antagonists, washed and then assessed in an MLR suppression assay. The addition of primed Treg clones into an MLR strongly inhibited allogeneic CD4^+^CD25^−^ T effector cell proliferation and IL-2 synthesis in a dose-dependent manner (Fig. 6A, C). The suppression of IL-2 synthesis was stronger when Treg clones were first primed in the presence of CD28 antagonists (Fig. 6C). The suppression of effector cells proliferation was also enhanced, although to a lesser extent (the suppression being already above 90% in the experimental conditions tested here; Fig. 6A). Priming of Treg clones in the presence of a CD28 antagonist also led to reduced production of IFN-γ, TNF-α and IL-6 cytokines (data not shown). We next tested whether the enhancement of suppression by CD28 antagonists could also be observed with polyclonal Treg from peripheral blood (thymus-derived Treg ot tTreg) instead of clonal Treg cells. We sorted CD4^+^CD25^high^CD127^low^ tTregs from human PBMCs, primed these Tregs with allogeneic mDCs and secondarily tested the Treg suppression of autologous CD4^+^CD25^−^ T effector cell stimulated with the same mDCs. First, it is important to note that clonal Tregs were far more effective at suppressing Teff cell proliferation than tTregs. This was already observed at high Treg:Teff ratio (72% suppression for tTregs vs. 99% for Treg#1 at a 1∶1 ratio) but was most evident at low ratios (Fig. 6B). Thus, Treg clones appear extremely potent at suppressing Teff cells in vitro, as evidenced by their ability to still induce >90% suppression of Teff proliferation at ratios of only 1 Treg per 100 or even per 1,000 Teff. Even more clearly than with Treg clones, priming of tTregs in the presence of CD28 antagonists improved the suppression of effector T cells proliferation and cytokine synthesis (Fig. 6B, D), indicating that our observations at the clonal level are relevant to the behavior of polyclonal tTregs.

## Discussion

Modification of T cell costimulation signals is becoming a therapeutic option in autoimmunity, transplantation and cancer. However, despite many advances in animal models, how costimulatory signals differentially control Teff and Treg reactivities as well as the consequences of costimulation blockade on Treg function and immune tolerance are not well defined in humans. We, and others, previously reported that costimulation blockade with selective CD28 antagonists resulted in suppression of auto- and allo-reactive Teff and induced Tregs and immune tolerance after organ transplantation in rodents and in primates [34], [41], [42], [43], [44], [45]. This suggested that Treg and Teff responses might be differentially controlled by costimulatory signals in humans. Owing to previously described differences between murine and human T cell molecular interactions, notably in the costimulation family of molecules [28], [46] and further considering the current clinical development of CD28 and CD80/86 antagonists to control autoimmunity and transplant rejection [29], [47], we aimed at studying the impact of costimulatory molecules interacting with CD80/86 [4] on human Teff and Treg functions. Using clonal cells representing alloreactive T cells that have already reacted with their antigen, we found that CD28, CTLA-4, and PD-L1 differentially control function and velocity, motility and immune synapse formation in Teff versus Treg and show for the first time in human cells that CTLA-4 does not reverse the TCR stop-signal in Treg, whereas it does in Teff. To our knowledge, it is also the first time that PD-L1-CD80 interactions are shown to suppress motility specifically in Treg and increases contact times in Teff.

A limitation of our study is that we used alloantigen-specific human Teff and Treg clones in order to be able to visualize cognate T-APCs interactions, an event that would occur at low frequency if polyclonal T cells were used. The reactivity of our cells might therefore not be representative of the general T cell population and might rather correspond to primed alloreactive T cells (i.e. cells that have already met antigen and have already proliferated). Indeed the Teff clone used here express CTLA-4 and therefore does not correspond to naïve T cells. We derived these clones from circulating CD3^+^CD4^+^CD25^−^ Teff and CD3^+^CD4^+^CD25^high^CD127^low^ Treg cells and selected single cells responding to allogeneic B-EBV cells in the presence of IL-2. Among 48 cell clones obtained from CD3^+^CD4^+^CD25^high^CD127^low^ cells, the Treg#1 clone was selected as one of two Foxp3+ clones that were consistently suppressive in vitro. Treg#1 clones were Foxp3^+^ Helios^−^ and presented a high suppressive activity in vitro. Of note, T cell clones did not express ICOS or ICOS-L (known to interact with CD28 in humans [28]) and the allogeneic B-EBV APCs expressed CD80 and CD86 but not ICOS-L, indicating that the ICOS/ICOS-L pathway was not at play in our system. The allogeneic B-EBV APCs did not express PD-1 and PD-L1 whereas both Teff and Treg clones expressed PD-L1 and at similar levels (as discussed below), suggesting that PD-L1 blockade may have disrupted selectively PD-L1 signals in T cells that resulted from interaction with CD80 on APCs. Thus, the major costimulatory and inhibitory interactions occurring between APCs and T cells in our system likely involve CD80/86 on APCs and CD28, CTLA-4 and PD-L1 expressed on T cells.

Our data show, for what we believe to be the first time, that CD80/86 ligands, namely CD28, CTLA-4 and PD-L1, control the early stages of interactions of some human Tregs with APCs. In particular, we found that blocking all or individual CD80/86 ligands altered to a different extent the duration of Treg contacts with APCs, motility of Tregs, calcium flux in response to antigen recognition and, ultimately, Treg suppressive function. Prolonged contacts of Tregs with APCs and formation of synapses required addition of a CD28 antagonist and availability of CTLA-4-CD80/86 interactions. Indeed we recorded incomplete but clear co-localization of PKC-θ and CD3 in Treg contacts with APCs under control conditions and after addition of CD28 antagonist mAbs (Fig. 2C, D). This result is contrasting with images from Zanin-Zhorov et al. who reported more pronounced altered formation of immune synapses in Treg and a localization of PKC-θ distal from the immune synapse and therefore not co-localized [37]. In addition, Zanin-Zhorov et al. reported on stable synapses with Treg cells, whereas with our conditions synapses were less stable with Treg than with Teff. This discrepancy may be due to important experimental differences, such as the use of clonal primed T cells here and in the use of supported planar bilayers containing anti-CD3 antibodies in the study by Zanin-Zhorov *et al* versus our model of induction of T-APCs contacts on a poly-L-lysin coat with cognate APCs. Recently, however, it was suggested that Treg synapses with DC were motile and frequently broke by the partial loss of pSMAC, suggesting that Treg might present a more dynamic behavior in comparison with Teff [48], which is more in relation with our current observations.

We found that CTLA-4 antagonist antibodies dampened the motility of human Teff but not Tregs, in agreement with recent data in mice [49]. We also show for the first time that PD-L1 antagonist antibodies increase the motility of human Tregs, suggesting that PD-L1 acts to reduce motility in these cells. It is likely that this occurs as a result of CD80 engagement with PD-L1 since PD-1, the primary ligand of PD-L1, is not expressed by the APCs used in the current study. Alternatively PD-L1 could interact with PD-1 expressed on other T cells (T-T interactions). Overall, we observed an opposite response of Teff and Tregs to signals transmitted by CD80/86 ligands, in that CD28 blockade resulted in the inhibition of Teff responses and the enhancement of Treg responses, in a CTLA-4-dependent and partially PD-L1-dependent manner. Experimentally, this translated into the enhancement of velocity and motility, the inhibition of immune synapse formation and the inhibition of calcium flux in Teff after CD28 blockade, whereas in Tregs we observed the enhancement of synapse formation and calcium flux without modification of motility.

Our study is supporting the emerging concept that CTLA-4 signals result in different outcomes, at least in some Teff and Tregs (such as the clons assessed here). Whether this is due to the use of differential signaling pathways in Teff and Tregs is currently under investigation by several groups. One possibility is that Rap1 and LFA-1, which plays a pivotal role in facilitating sustained T cell–APC adhesion [50], are differentially regulated by CD28 and CTLA-4 in Tregs and Teff. The current model holds that TCR activation of Rap1, which is inhibited by CD28 and activated by CTLA-4, induces (inside-out) changes in conformation and increases the affinity of LFA-1 [51], [52]. For Tregs, this is in agreement with our data showing that CD28 signals decreased whereas CTLA-4 signals increased adhesion (in other words, that CD28 antagonists increased whereas CTLA-4 antagonists decreased adhesion). However, this interpretation was not confirmed by direct analysis of LFA-1 since inhibition of costimulatory signals did no induce any change in LFA-1 conformation in Tregs. In Teff cells, we observed that the ratio of activated/total CD18 was reduced after CD28 blockade and increased after CTLA-4 blockade. These data suggest differences in the CD28/CTLA-4/LFA-1 axis between the Teff and Treg cells analyzed here.

In Teff cells expressing CTLA-4, we found that contact times with APCs were directly regulated by CD28 antagonist antibodies but that this occurred in a strictly CTLA-4-dependent manner. In conditions where both CD28 and CTLA-4 are available for interacting with CD80/86, CD28 appears to be dominant over CTLA-4 and therefore prevents CTLA-4 from blocking the TCR-mediated stop signal, resulting in high dwell times in Teff cells. Concerning Treg cells, It was previously reported in mice that CTLA-4 does not block the TCR mediated stop signal and does not control Treg dwell time [49]. Here, we observed that the dwell time, but not motility, of human Tregs was controlled by CD28 and CTLA-4. Indeed, upon CD28 blockade, we observed Tregs establishing longer contacts with APCs but then moving together with the APC in a paired type of movement without any reduction in overall Treg motility. We could not detect similar movements of Teff-APC pairs, suggesting that this phenomenon is limited to human Tregs. Of note, this type of movements may be visible more readily in our model than in other systems because cells were allowed to move freely on the microscope slides in the poly-L-lysin coat in our study, without addition of immobilized ICAM-1. Since in vivo two-photon laser-scanning microscopy cannot be performed in humans, our model system may thus best mimic free T-APC interactions and movements as they occur in vivo. Blockade of CTLA-4 increased Treg velocity (Fig. 3J), indicating a direct role for CTLA-4 in slowing down Tregs. We also identified a role for PD-L1 in controlling human Treg motility (Fig. 2I) but concomitant PD-L1 blockade had no further effect on Treg dwell times over conditions where CD28 and CTLA-4 were blocked. Overall, our data therefore suggest that the suppressive function of Tregs is correlated with contacts with APCs rather than motility per se since we noticed enhanced contact times and suppressive function in the presence of CD28 antagonists without modification of motility and velocity parameters.

Lu et al reported no difference in contact time, motility and displacements between CD4^+^ T cells from CD28^+/+^ and CD28^−/−^ mice, assigning a role of CD28 only to signaling events in already formed T cell-DC conjugates [49]. In contrast, using antagonist Abs and human T cells, we observed a role for CD28 in Teff velocity, dwell time and motility (although this parameter is a direct consequence of modification of cell adhesion), and in Treg dwell time and suppressive function. This discrepancy may be due to different biology of Treg cells in mice versus humans that has been attested by the differential function of several molecular interactions between the two species [28], [46]. Moreover, it could be related to the use of CD28 antagonists vs. genetic inactivation of CD28, as the latter may have resulted in the selection of Treg cells functioning in a CD28-independent manner, as suggested previously [53]. Finally, as previously stated, our T cell clones are not representative of cells in their naïve state and rather correspond.

The fact that CD28 signals dampen suppression by Treg cells [17] and, as a correlate, that CD28 blockade amplifies Treg suppression, as shown here, finds an explanation in that CD28 induces activation of AKT, which in turn inhibits Foxo1 and Foxo3 transcription factors that are required for the suppressive function of Tregs [18], [19], [20]. CD28, however, is important for the thymic generation of Tregs and their peripheral homeostasis [16], [54], [55]. Therefore, it is possible that blockade of CD28-mediated signals in vivo will result in enhancement of the suppressive activity of Tregs but also a reduction in their generation and number. Indeed, several studies reported an overall decrease in peripheral Tregs in kidney transplant recipients or rheumatoid arthritis patients treated with Nulojix® or Orencia®, which are CTLA4-Ig molecules binding CD80/86 and preventing CD28-mediated costimulation [56], [57], [58]. However, an expansion of Treg cells has also been recorded in experimental models of transplantation where recipients were treated with selective CD28 antagonists [34], [44], [45], suggesting that either some Tregs can expand independently of CD28. In this regard, CD28-independent Treg cells depending on ICOS costimulation have recently been described [53].

**Figure 7:**
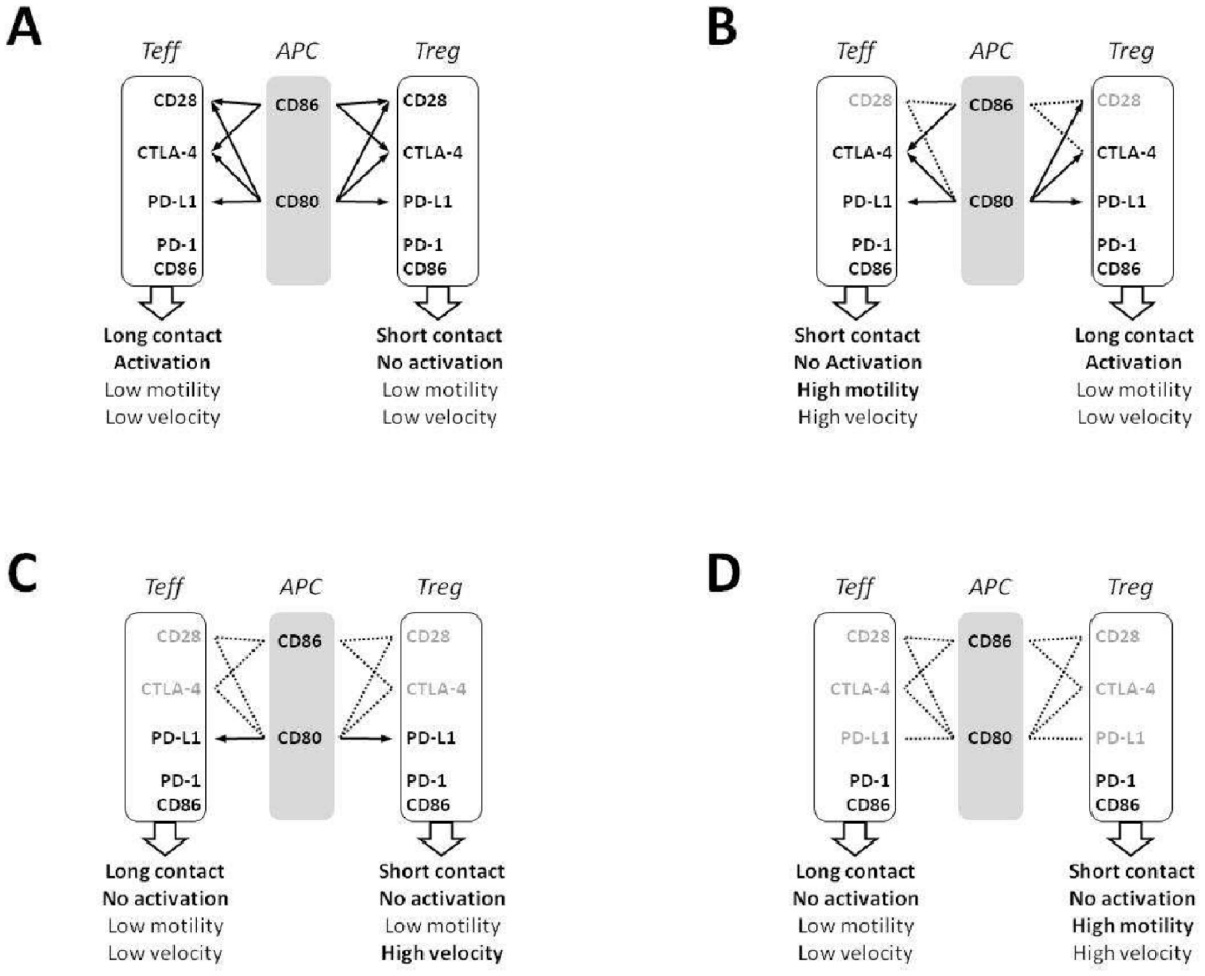
Proposed model for the role of CD28, CTLA-4 and PD-L1 in human Teff and Treg-APCs interactions. In control conditions, CD80/86 expressed by APCs interact with CD28, CTLA-4 and PD-L1 on Teff and Treg leading to long contacts and activation of Teff and to short contacts and absence of activation of Treg (A). In the presence of a selective CD28 antagonist, CD80/86 interact with CTLA-4 and PD-L1, leading to enhanced motility, reduced contacts and absence of activation in Teff, and to enhanced contacts and activation in Treg. Interestingly Treg motility is not affected, showing absence of modulation by CTLA-4 of the TCR-Stop signal in human Treg (B). If CD28 and CTLA-4 are simultaneously blocked, CD80 interacts with PD-L1. In these conditions Teff make long contacts with APCs but do not activate. Treg make short contacts and do not activate either, like in control conditions, and show enhanced velocity but not motility (C). If CD28, CTLA-4 and PD-L1 are simultaneously blocked, the only additional difference is that motility is increased in Treg, which shows that PD-L1 controls motility of human Treg (D). PD-1 and CD86 are also expressed by T cells and this adds a layer of complexity if T-T interactions had to be also addressed in addition to T-APCs interactions.

In conclusion, we have demonstrated that antagonist antibodies against CD28, CTLA-4 and PD-L1 differentially regulated interactions of primed alloreactive human Teff and Treg clones with APCs, resulting in mirror-image outcomes in these cell subsets (modelized in Fig. 7). In human Teff, CD28 promoted while CTLA-4 inhibited T-APCs interactions, synapse formation and cell activation. PD-L1, possibly by interacting with CD80, synergized with CTLA-4 to minimize T-APCs contacts. In contrast, in Tregs, CD28 inhibited and CTLA-4 promoted synapse formation and cell activation, which correlated with their suppressive function. A novel role for PD-L1 consisting in reducing the motility of human Tregs was also identified in our study. Thus, individually targeting CD28, CTLA-4 and PD-L1 with biological agents might represent a valuable therapeutic strategy to treat immune disorders where Teff and Treg functions need to be differentially targeted.

## Supporting Information

Movie S1 **Human Teff form long lasting contacts with APCs, in control condition.** Representative time-lapse video of human Teff cells stained with Fura-2AM (fluorescent calcium probe), incubated at 37°C with unstained APCs (human EBV-B lymphoblastoid cells). Cells were added on 0.001% poly-L-lysine coated Lab-Tek chambers and images were taken every 15 sec over 25 minutes. Non-activated Teff (green fluorescence) become red after increase of calcium concentration, thus demonstrating activation. Average contact-time, motility and velocity are shown in Fig. 3A, B, E, F and G. Calcium responses are shown in Fig. 4A and B. Time is shown in minutes.
(MOV)

Movie S2 **CD28 antagonists break Teff–APC contacts.** Representative time-lapse video similar to Movie S1 (over 25 minutes), performed in the presence of 10 µg/ml FR104, an antagonist anti-CD28 antibody. Teff (green) establish few contacts and present low calcium fluxes demonstrating absence of activation. Contact-time, motility are shown in Fig. 3A, B, E, F and G. Calcium responses are shown in Fig. 4A and B.
(MOV)

Movie S3 **Addition of CTLA-4 antagonists to CD28 antagonists restores Teff–APC contacts but not activation.** Representative time-lapse video similar to Movie S1 (over 25 minutes), performed in the presence of 10 µg/ml FR104, an antagonist anti-CD28 antibody plus 10 µg/ml 147.1, an antagonist anti-CTLA-4 antibody. Teff (green) dwell on APCs but do not show activation. Contact-time, motility are shown in Fig. 3A, B, E, F and G. Calcium responses are shown in Fig. 4A and B.
(MOV)

Movie S4 **Human Treg form short contacts with APCs, in control condition.** Representative time-lapse video of human Treg cells stained with Fura-2AM (fluorescent calcium probe), incubated at 37°C with unstained APCs (human EBV-B lymphoblastoid cells). Cells were added on 0.001% poly-L-lysine coated Lab-Tek chambers and images were taken every 15 sec over 25 minutes. Treg (green) show weak basal calcium fluxes. Contact-time, motility are shown in Fig. 3C, D, H, I and J. Calcium responses are shown in Fig. 4C and D.
(MOV)

Movie S5 **CD28 antagonists induce long lasting contacts between human Treg and APCs.** Representative time-lapse video similar to Movie S4 (over 25 minutes), performed in the presence of 10 µg/ml FR104, an antagonist anti-CD28 antibody. Treg (green) become red showing an increase of intracellular calcium flux and thus Treg activation. Contact-time, motility are shown in Fig. 3C, D, H, I and J. Calcium responses are shown in Fig. 4C and D.
(MOV)

Movie S6 **Addition of CTLA-4 antagonists to CD28 antagonists restores Teff–APC short contacts between Treg and APCs.** Representative time-lapse video similar to Movie S4 (over 25 minutes), performed in the presence of 10 µg/ml FR104, an antagonist anti-CD28 antibody plus 10 µg/ml 147.1, an antagonist anti-CTLA-4 antibody. Treg (green) showed low levels of calcium flux. Contact-time, motility are shown in Fig. 3C, D, H, I and J. Calcium responses are shown in Fig. 4C and D.
(MOV)
